# Supplementary material for: Beyond personal factors: Multilevel determinants of childhood stunting in Indonesia
Source: PLoS One. 2021 Nov 19;16(11):e0260265. doi: 10.1371/journal.pone.0260265 (PMC8604318; doi:10.1371/journal.pone.0260265)
Supplement: S1 Appendix — (DOCX) [file pone.0260265.s001.docx]

S1 Appendix

Table Appendix 1. Results of Stepwise Multilevel Mixed Effects Logistic Regression Models

|  | **Child level covariates** | | | **Family/household covariates** | | | **Community covariates** | | | | |
| --- | --- | --- | --- | --- | --- | --- | --- | --- | --- | --- | --- |
| Stunted | **Model 1**  **Odds Ratio**  **(95% CI)** | **Model 2**  **Odds Ratio**  **(95% CI)** | **Model 3**  **Odds Ratio**  **(95% CI)** | **Model 4**  **Odds Ratio**  **(95% CI)** | **Model 5**  **Odds Ratio**  **(95% CI)** | **Model 6**  **Odds Ratio**  **(95% CI)** | **Model 7**  **Odds Ratio**  **(95% CI)** | **Model 8**  **Odds Ratio**  **(95% CI)** | **Model 9**  **Odds Ratio**  **(95% CI)** | **Model 10**  **Odds Ratio**  **(95% CI)** | **Model 11**  **Odds Ratio**  **(95% CI)** |
| Gender  *Male* | 1.15** (1.02-1.30) | 1.17***  (1.05-1.32) | 1.16**  (1.03-1.31) | 1.16**  (1.03-1.31) | 1.16**  (1.03-1.32) | 1.17**  (1.04-1.32) | 1.17**  (1.03-1.31) | 1.17***  (1.04-1.32) | 1.17***  (1.04-1.32) | 1.17***  (1.04-1.32) | 1.17**  (1.04-1.32) |
| Baby size  *Small baby* |  | 2.41***  (1.83-3.18) | 2.43***  (1.84-3.20) | 2.44***  (1.85-3.23) | 2.30***  (1.74-3.03) | 2.33***  (1.76-3.06) | 2.31***  (1.76-3.04) | 2.31***  (1.76-3.04) | 2.31***  (1.76-3.05) | 2.30***  (1.75-3.03) | 2.40***  (1.81-3.18) |
| Diarrhea  *Acute diarrhea (3 times/day in past 4 weeks)* |  |  | 1.30***  (1.11-1.53) | 1.30***  (1.10-1.38) | 1.29***  (1.09-1.51) | 1.31***  (1.12-1.54) | 1.32***  (1.13-1.55) | 1.32***  (1.13-1.56) | 1.32***  (1.12-1.55) | 1.30***  (1.11-1.53) | 1.29***  (1.10-1.52) |
| Mother’s stature  *Mother short (<145 cm)* |  |  |  | 1.22***  (1.08-1.38) | 1.25***  (1.10-1.41) | 1.22***  (1.08-1.38) | 1.22***  (1.08-1.38) | 1.22***  (1.08-1.38) | 1.22***  (1.08-1.38) | 1.22***  (1.08-1.38) | 1.21***  (1.07-1.37) |
| Mother’s education |  |  |  |  | 0.93***  (0.24-0.42) | 0.95***  (0.93-0.97) | 0.95***  (0.94-0.97) | 0.95***  (0.94-0.97) | 0.96  (0.94-0.97) | 0.96***  (0.95-0.98) | 0.96***  (0.95-0.99) |
| Consumption quartile  *2^nd^ quartile*  *3^rd^ quartile*  *4^th^ quartile* |  |  |  |  |  | 0.74***  (0.63-0.88)  0.68***  (0.57-0.81)  0.49***  (0.40-0.60) | 0.75***  (0.63-0.88)  0.69***  (0.58-0.82)  0.50***  (0.41-0.63) | 0.75***  (0.64-1.17)  0.70***  (0.58-0.83)  0.51***  (0.42-0.62) | 0.76***  (0.65-0.90)  0.72***  (0.60-0.85)  0.53***  (0.43-0.64) | 0.76***  (0.65-0.90)  0.72***  (0.60-0.86)  0.54***  (0.45-0.66) | 0.76***  (0.64-0.90)  0.72***  (0.60-0.86)  0.53***  (0.44-0.65) |
| Regional differences  *Rural* |  |  |  |  |  |  | 1.37***  (1.18-1.44) | 1.36***  (1.17-1.59) | 1.32***  (1.13-1.54) | 1.17*  (1.00-1.37) | 1.17*  (1.00-1.38) |
| Clean water  *No access* |  |  |  |  |  |  |  | 1.36*  (0.98-1.89) | 1.31  (0.94-1.82) | 1.31  (0.95-1.82) | 1.33*  (0.95-1.85) |
| Sanitation  *No access* |  |  |  |  |  |  |  |  | 1.23***  (1.07-1.42) | 1.20**  (1.04-1.38) | 1.19**  (1.03-1.38) |
| Hygiene  *No access* |  |  |  |  |  |  |  |  |  | 1.47***  (1.24-1.75) | 1.45***  (1.22-1.73) |
| Nutrition services  *No access* |  |  |  |  |  |  |  |  |  |  | 1.06  (0.88-1.26) |
| *Intercept* | 0.19  (0.15-0.25) | 0.19  (0.14-0.24) | 0.18  (0.14-0.23) | 0.16  (0.12-0.21) | 0.32  (0.24-0.42) | 0.39  (0.30-0.51) | 0.33  (0.25-0.44) | 0.32  (0.24-0.43) | 0.29  (0.22-0.39) | 0.23  (0.17-0.31) | 0.23  (0.16-0.32) |
| *Province effect – coef (std.dev)* | 0.14  (0.08) | 0.14  (0.09) | 0.14  (0.086) | 0.14  (0.08) | 0.13  (0.08) | 0.11  (0.06) | 0.11  (0.06) | 0.11  (0.06) | 0.11  (0.06) | 0.12  (0.07) | 0.11  (0.06) |
| *Subdistrict effect – coef (std.dev)* | 0.46  (0.08) | 0.46  (0.08) | 0.45  (0.08) | 0.46  (0.08) | 0.33  (0.07) | 0.29  (0.06) | 0.24  (0.06) | 0.23  (0.06) | 0.22  (0.06) | 0.20  (0.05) | 0.20  (0.06) |
| *Household effect – coef (std.dev)* | 0.80  (0.21) | 0.77  (0.21) | 0.79  (0.21) | 0.79  (0.21) | 0.82  (0.21) | 0.76  (0.21) | 0.76  (0.20) | 0.77  (0.21) | 0.75  (0.20) | 0.74  (0.20) | 0.76  (0.21) |
| *Observations* | 8105 | 8105 | 8105 | 8105 | 8045 | 8045 | 8045 | 8045 | 8045 | 8045 | 7795 |
| - For Model 1-4, we analyzed data from 8105 children who met the inclusion criteria with anthropometric measures within biologically plausible values. - For Model 5-10, we analyzed data from 8045 children who met the inclusion criteria with anthropometric measures within biologically plausible values and with complete individual and household characteristics. - For Model 11, we analyzed data from 7795 children who met the inclusion criteria with anthropometric measures within biologically plausible values and with complete individual and household characteristics as well as information on nutritional services access. However, this model was not used in the following estimation due to insignificant findings. - An odds ratio was statistically significant at either 1 percent (***), 5 percent (**) or 10 percent (*) of the confidence intervals. | | | | | | | | | | | |
