# Supplementary material for: Beyond personal factors: Multilevel determinants of childhood stunting in Indonesia
Source: PLoS One. 2021 Nov 19;16(11):e0260265. doi: 10.1371/journal.pone.0260265 (PMC8604318; doi:10.1371/journal.pone.0260265)
Supplement: Attachment — Submitted filename: Reviewed May 2021 Stunting in Indonesia.docx [file pone.0260265.s002.docx]

May 30, 2021

Reviewer’s comments

Manuscript title: Beyond Personal Factor: Multilevel Determinants of Childhood Stunting in Indonesia

Manuscript Number: PONE-D-21-06348

Dear editor, thank you so much for inviting me to review this manuscript. Here are my comments.

**Abstract section:**

- Not structured

**Introduction section:**

- Authors mixed up reference citation styles (Vancouver vs Harvard styles) - see paragraph 1, lines 9-10.

**Methods section:**

- The authors have mentioned that there were 4,309 children in wave 4 and 4,235 children in wave 5 who were less than five years old. However, they also mentioned that there were 5,975 households who had children aged 5 and below. How can the number of households who had children under five be more than the number of children age less than five? Does that mean some of the children did belong to more than one households?
- The authors need to elaborate the following issues:
  - Which WHO curve was used to determine the nutritional status (stunting) of the children? The 2006 or….?
  - Who determined the nutritional status (stunting), the data collectors at the time of the data collection or the authors of this manuscript (by analyzing the raw data i.e. height-to-age of the child)?
- The community level variable that is “access to nutrition specific services” lacks clarity. The authors categorized the variable based on the accessibility of a child to three nutrition specific services provided by PHC in the area. What does “access” means? Does it necessarily imply the utilization of the mentioned services? How can a child be categorized if those mentioned services are available but child is not utilizing any of the services?
- I am not sure if it is necessary to describe the details of the mathematical formulas that were used by the authors to run the different prediction models as it is done by the authors of this manuscript. Because of such lengthy description, it is no attractive for reading. Better only to state the models and why those models are preferred.
- Section 2.7 describes the authors’ findings. But it is kept under the “Data and Methods” section. Better to move it to the “Result section” as section 2.7 contains the research findings not research method.

**Section 2.7. characteristics of study participants:**

- Table 1: Standard deviation the variable “stunted” is mentioned as 0.440. How can a categorical variable have a standard deviation?
- Table 1 is confusing and difficult to understand because of the following reasons:
  - The purpose of calculating “standard deviation” for categorical variables is not clear.
  - Look at the following two observations which are directly taken from the table ( similar problem exists for other variables too):
    - Gender
      - Male= 8105
      - Female= 0
    - Baby size
      - Small baby= 8105
      - Normal baby=0

From the above observations, what readers can understand is that there were no female babies and normal size babies.

- Table 4: The table cells contain “starred” values. What do those “stars” imply? Needs description as foot note under the table.
- In the methods section the authors mentioned that there were 4,309 children in wave 4 and 4,235 in wave 5 of the survey. However, in the result section they mentioned that they have included 4015 children of wave 4 and 4090 children of wave 5 (Total of 8105 children). The authors need to explain how and why some of the children were excluded from the analysis.
- The total number of children included in the analysis is 8105 (the sum of children in wave 4 and wave 5). It is not clear why the authors summed up the number of children from the two waves and treated the two group of populations as single population. The population of children in wave 4 and wave 5 are separate since data collected from the two group of population was collected at different times (2007 and 2014) and should not be summed up and should not be treated as homogenous population.
- Result and discussion are mixed. I advise the authors to follow the journal’s template.
- Authors need to mention the limitation of their study.

**Conclusion:**

- Under this section, the authors tried to summarize their manuscript rather than putting the main finding of their research. Better to revise this section in such a way.

Thank you
