# Supplementary material for: Beyond personal factors: Multilevel determinants of childhood stunting in Indonesia
Source: PLoS One. 2021 Nov 19;16(11):e0260265. doi: 10.1371/journal.pone.0260265 (PMC8604318; doi:10.1371/journal.pone.0260265)
Supplement: Attachment — Submitted filename: Response to Reviewers.docx [file pone.0260265.s004.docx]

**Responses to Reviewers**

Article titled “Beyond Personal Factors: Multilevel Determinants of Childhood Stunting in Indonesia”

| **No** | **Comments to the Author** | **Responses to reviewers’ comments** |
| --- | --- | --- |
| 1 | If the authors have adequately addressed your comments raised in a previous round of review and you feel that this manuscript is now acceptable for publication, you may indicate that here to bypass the “Comments to the Author” section, enter your conflict of interest statement in the “Confidential to Editor” section, and submit your "Accept" recommendation.  Reviewer #3: (No Response)  Reviewer #4: (No Response) | We have revised the manuscript according to editor and reviewers’ inputs as described below. |
| 2 | Is the manuscript technically sound, and do the data support the conclusions?  The manuscript must describe a technically sound piece of scientific research with data that supports the conclusions. Experiments must have been conducted rigorously, with appropriate controls, replication, and sample sizes. The conclusions must be drawn appropriately based on the data presented.  Reviewer #3: Yes  Reviewer #4: Partly | Thanks for your comment. We have revised the conclusion as per reviewer #4 suggestion in point 6 below. |
| 3 | Has the statistical analysis been performed appropriately and rigorously?  Reviewer #3: Yes  Reviewer #4: Yes | Thanks for your comment. |
| 4 | Have the authors made all data underlying the findings in their manuscript fully available?  The [PLOS Data policy](http://www.plosone.org/static/policies.action#sharing) requires authors to make all data underlying the findings described in their manuscript fully available without restriction, with rare exception (please refer to the Data Availability Statement in the manuscript PDF file). The data should be provided as part of the manuscript or its supporting information, or deposited to a public repository. For example, in addition to summary statistics, the data points behind means, medians and variance measures should be available. If there are restrictions on publicly sharing data—e.g. participant privacy or use of data from a third party—those must be specified.  Reviewer #3: Yes  Reviewer #4: Yes | Thanks for your comment. |
| 5 | Is the manuscript presented in an intelligible fashion and written in standard English?  PLOS ONE does not copyedit accepted manuscripts, so the language in submitted articles must be clear, correct, and unambiguous. Any typographical or grammatical errors should be corrected at revision, so please note any specific errors here.  Reviewer #3: Yes  Reviewer #4: No | The manuscript has been proof read by American Journal Experts to assure that the manuscript is presented in an intelligible fashion, clear, correct, unambiguous and have no grammatical errors. |
| 6 | Review Comments to the Author  Reviewer #3: All comments are addressed properly. The manuscript is technically sound, statistical analysis has been performed appropriately and rigorously and manuscript is presented in an intelligible fashion but need some grammatical corrections.  Reviewer #4:  1. The manuscript is unnecessarily long such that the core issues are masked 2. The methodology section need not include detailed formulae used in deriving the results. This section needs revising to make it shorter and succinct  3. The results tables are too crowded. Summarised tables can be given in the results and more elaborate tables provided as addendum, if necessary  4. The conclusions read like another discussion of results. This section needs to be focussed on answering the objectives of the study | The manuscript has been proof read by American Journal Experts to assure that the manuscript is presented in an intelligible fashion, clear, correct, unambiguous and have no grammatical errors.  Regarding reviewer#4 input number 1 and 2, we have removed the empirical model in the methodology section and make the section shorter. Hopefully, the revised manuscript is more concise.  Thanks for the suggestion. We relocated table 3 of stepwise results into appendix.  Thanks for the suggestion. We have revised the conclusion so it is more focus on answering the objectives of the study. Moreover, we relocated the study limitation from conclusion into discussion. We added a subsection at the end of the discussion section to explain the study limitation. |
| 7 | PLOS authors have the option to publish the peer review history of their article ([what does this mean?](https://journals.plos.org/plosone/s/editorial-and-peer-review-process#loc-peer-review-history)). If published, this will include your full peer review and any attached files.  If you choose “no”, your identity will remain anonymous but your review may still be made public.  **Do you want your identity to be public for this peer review?** For information about this choice, including consent withdrawal, please see our [Privacy Policy](https://www.plos.org/privacy-policy).  Reviewer #3: No  Reviewer #4: No | Yes, noted. |
| 8 | Despite the known influence of the dietary behaviours and habits on growth and the effect of the national school feeding programs on improving stunting among school children, yet the introduction and discussion lack a lot of references concerning national figures for stunting and its determinants among children in low- and middle-income countries with similar context. Commenting on the impact of the national school Feeding programs on growth and subsequently on development and school achievement as a solution and remedy way for improving stunting in the discussion section (which is vital) is still missing. Please elaborate on this in structural relationships within the discussion, and recommendations. | Thanks for the suggestion.   - We have added a paragraph in the introduction presenting data concerning figures for stunting among children in low- and middle-income countries with similar context. - We have added citation of articles discussing the effect of the national school feeding programs on improving nutritional status among school children in the discussion section to explain the role of dietary habits on children nutritional status. - There are three additional literatures that we have added to explain the importance of dietary habits in lowering risk of nutritional issues such as stunting as below.  1. Metwally AM, El-Sonbaty MM, el Etreby LA, Salah El-Din EM, Abdel Hamid N, Hussien HA, et al. Impact of National Egyptian school feeding program on growth, development, and school achievement of school children. World Journal of Pediatrics. 2020 Aug 13;16(4). 2. Murphy SP, Gewa C, Liang L-J, Grillenberger M, Bwibo NO, Neumann CG. School Snacks Containing Animal Source Foods Improve Dietary Quality for Children in Rural Kenya. The Journal of Nutrition. 2003 Nov 1;133(11). 3. Baum JI, Miller JD, Gaines BL. The effect of egg supplementation on growth parameters in children participating in a school feeding program in rural Uganda: a pilot study. Food & Nutrition Research. 2017 Jan 6;61(1). |
